# Study design considerations for mandibular and maxillary research in the OVX rat model

**DOI:** 10.3389/fdmed.2026.1968183

**Published:** 2026-10-27

**Authors:** Mitchell A. Ianiero, Wendy E. Ward

**Affiliations:** 1 Department of Health Sciences, Faculty of Applied Health Sciences, Brock University, St. Catharines, ON, Canada; 2 Department of Kinesiology, Faculty of Applied Health Sciences, Brock University, St. Catharines, ON, Canada

**Keywords:** bone mineral density, bone structure, mandible, maxilla, methodology, ovariectomy, rats

## Abstract

A recent systematic review (SR) and meta-analysis reporting the changes in BMD and bone structure in the mandible post-ovariectomy (OVX) noted high heterogeneity among the primary studies. This perspective addresses aspects in study design that should be considered and reported to reduce heterogeneity in future OVX studies investigating mandible and maxilla BMD and bone structure. A search of the literature conducted in Medline via OVID for studies analyzing BMD or bone structure in the mandible and maxilla of rats post-OVX identified 43 studies for inclusion. This perspective discusses and shares recommendations for five aspects within a study that, if more closely controlled and/or reported within a publication, can contribute to more meaningful comparisons of findings among studies using the OVX rat model to investigate changes in mandible and maxilla due to OVX or in response to an intervention. These aspects include: age at time of OVX; time since OVX; feeding protocol; diet texture and nutrient composition; and type of imaging use. Following these recommendations can help inform future study design and allow for more direct comparisons among studies. In turn, findings from such individual studies can be assessed within SRs that include meta-analyses and enhance an understanding of using the OVX rat model in this field of research.

## Introduction

The ovariectomized (OVX) rat model is widely used and considered the gold standard model for studying post-menopausal osteoporosis and testing potential interventions (1, 2). It involves the surgical removal of the ovaries to induce a loss of ovarian estrogen production that stimulates an increased rate of bone turnover and bone resorption (2, 3). The characteristic loss of bone mineral density (BMD) and deterioration of bone structure occurring at multiple skeletal sites in the OVX rat resembles that which occurs in post-menopausal females (3–5). The OVX rat model also mimics other aspects reported in women: an initial rapid phase of bone loss followed by a slower phase; a greater loss of trabecular bone compared to cortical bone; decreased intestinal absorption of calcium; and a similar response to pharmacotherapy, nutrition or exercise interventions (3, 4, 6–11).

Loss of alveolar bone in the mandible and maxilla can occur in post-menopausal women and contribute to tooth loss (5, 12–14). In turn, tooth loss can cause difficulties eating whole foods (e.g., fruit, vegetables), leading to a poor diet and increased risk of chronic disease (15, 16). Moreover, changes in BMD or bone structure in the alveolar bone region of the mandible and maxilla in the OVX rat reflect the loss of BMD and deterioration of bone structure at the lumbar vertebrae, femur, and tibia (17–22). Thus, using the OVX rat model to assess potential interventions that can preserve skeletal health at multiple skeletal sites, including the alveolar bone in the mandible, is of interest.

A recent systematic review (SR) and meta-analysis examined the changes in BMD and bone structure in the mandible of OVX rats. This SR concluded that BMD, bone volume fraction (BV/TV), and trabecular thickness (Tb.Th) was significantly lower and trabecular separation (Tb.Sp) was significantly higher in OVX compared to SHAM rats (1). However, this study found high heterogeneity in the analyses that limited the reliability of the pooled effects and thus the ability to draw definitive conclusions from the meta-analyses. The authors attributed the heterogeneity to two main factors: the varying durations of time since OVX and the region of interest (ROI) in the mandible (1). Other contributors to heterogeneity may include the age of the rat at OVX (i.e., growing), the feeding regimen (i.e., *ad libitum* versus pair-feeding), diet texture and composition, and the type of imaging performed (i.e., dual energy x-ray absorptiometry or micro-computed tomography).

This perspective discusses how these factors—embedded in study design—may contribute to heterogeneity among studies that investigate changes in BMD or bone structure in the mandible or maxilla post-OVX. The objective of this perspective was to identify and discuss a few key aspects that should be considered, controlled, and reported among these types of studies to minimize heterogeneity. By reducing this heterogeneity, meta-analyses within SRs can lead to more conclusive findings, to best inform the design of future studies investigating interventions that support alveolar bone health and tooth retention using the OVX rat model. This perspective also contributes to the broader call for improving reproducibility through the ARRIVE guidelines (Animal Research: Reporting of *in vivo* Experiments) (23). The ARRIVE guidelines are in place to allow researchers to evaluate methodological rigor and reproduce study findings (23).

## Search strategy

A literature search was conducted in Medline via OVID (published from 1990 to present) using (ovariectomy OR ovariectomized) and (rat OR rats) and (“bone mineral density” OR “bone structure” OR “bone microarchitecture” OR “alveolar bone” OR “bone loss”) and (mandible OR mandibular OR maxilla) on February 13, 2024 with a repeat search on July 27, 2026 to identify studies published after the first search was conducted. Inclusion criteria included the following: studies with at least one outcome of BMD or bone structure measured in the mandible or maxilla in OVX and SHAM rats; studies with a nutritional or pharmacological intervention that included both a SHAM and OVX group that did not receive the intervention; and studies published in the English language. Exclusion criteria included studies published prior to 1990 and studies with a sample size of <5 rats per group for either the SHAM and/or OVX groups. The two searches identified a total of 141 records. Thirty-eight studies satisfied the inclusion and exclusion criteria and an additional 5 studies, which also satisfied the inclusion and exclusion criteria, were identified from references of papers reviewed. Forty-three studies that included outcomes of BMD and/or bone structure in the rat mandible and/or maxilla post-OVX and SRs of such studies with or without meta-analyses were reviewed. This includes one SR with a meta-analysis which included 18 primary studies, of which 16 were included in this perspective; two of the primary articles were not included due to a small sample size (*n* = 3) in the OVX and SHAM groups (1).

### Age at time of OVX

By age 6 months, it is known that femur and tibia bone growth has slowed and suggests 6 months as an ideal age for OVX (3, 14). By approximately 9 months of age, rats experience age-related bone loss at multiple skeletal sites (proximal tibia, femur, lumbar vertebrae), such that the OVX surgery is difficult to separate from the effect of aging (4, 9, 11). Less studied is whether growth and aging interfere with the characteristic loss of BMD and deterioration of bone structure at the mandible or maxilla expected with OVX. However, review of the literature identified a loss of BMD and deterioration of bone structure at various sites of the mandible and maxilla in most studies, suggesting that growth and aging may have a lesser effect on these skeletal sites compared to the spine and long bones.

Most studies of the alveolar region of the mandible found a loss of BMD and deterioration of bone structure that is consistent with the known effect of OVX compared with SHAM rats (Supplementary Tables S1 and S2) (20, 24–31). This occurred regardless of whether rats were young (<6 months of age) or skeletally mature (6–9 months of age) at the time of OVX. At other sites within the mandible, the effect of age at time of OVX is less clear and may be due to a lesser response of cortical bone. Regarding cortical bone alone at various sites, there were mixed findings in both young rats (cortical thickness, mineral-to-matrix ratio) as well as skeletally mature rats (tissue mineral density, cortical BMD, bone area, and/or thickness). Of note is the small number of studies studying cortical bone and the wide range of study durations post-OVX. Among studies of the mandibular body (trabecular and/or cortical bone) and mandibular condyle in young rats, several studies found no difference in BMD and/or bone structure between OVX and SHAM rats (20, 32–35). This may be due to effects of rapid growth during this stage of life, which may mask the effects of OVX. In contrast, most studies in skeletally mature rats found loss of BMD and deterioration of bone structure consistent with OVX at the mandibular body (trabecular and/or cortical bone) and mandibular condyle (36–41). This may be due to age-related bone loss that occurs with increased age at the time of OVX, which exacerbates the effects of OVX.

In summary, age at time of OVX did not appear to influence the effect of OVX on BMD or bone structure in the mandible, particularly in the alveolar region. However, to reduce heterogeneity across studies and based on known effects of aging on BMD and bone structure at other skeletal sites post-OVX (long bones, spine), it is likely best practice to follow the existing guidance of performing OVX surgery at 6 months of age (Figure 1).

**Figure 1:**
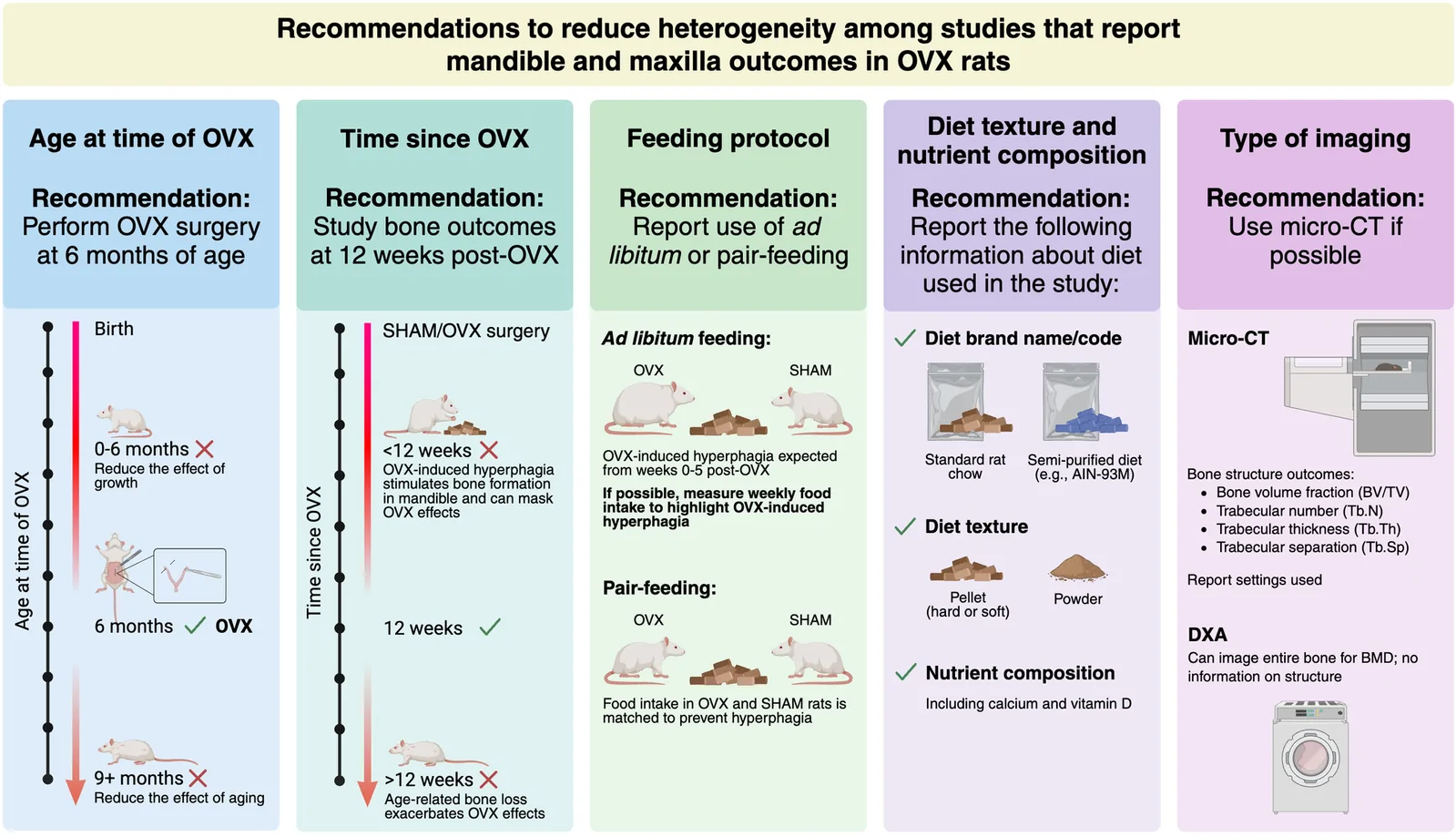
Aspects of study design that may contribute to heterogeneity among studies measuring BMD and bone structure in the mandible or maxilla post-OVX (Created with BioRender.com).

### Time since OVX

The duration of the study period post-OVX is another consideration that is variable across studies, and depending on the feeding protocol used, it may influence bone outcomes due to hyperphagia. OVX can induce a transient hyperphagia when food is provided *ad libitum* and can stimulate weight gain (42). In a study of 3-month-old Sprague-Dawley rats fed *ad libitum*, food intake was significantly higher in OVX rats compared to SHAM rats for the first 5 weeks post-OVX but was similar between groups from weeks 6–14 (42). Similarly, Sprague-Dawley rats that were OVX at age 6 months had a higher food intake than SHAM rats from weeks 2–3 with a similar intake from weeks 4 through 12 post-OVX (43). Thus, food consumption in rats increased in the first few weeks following OVX surgery and stabilized over time. The higher food consumption may affect mandible due to the force produced by masticatory muscles during chewing, which can stimulate bone formation and suppress bone resorption in the mandible (34, 44). Therefore, increased food consumption due to OVX early in a study may stimulate bone formation and counteract against a loss of BMD and deterioration of bone structure due to estrogen deficiency. Depending on the duration of an intervention post-OVX, BMD and bone structure may be influenced by higher food intake and associated force/loading on mandible. Among the studies reviewed and summarized in Supplementary Tables S1 and S2, there is a lack of consistency in study duration post-OVX. Because a significant loss of BMD and deterioration of bone structure by 12 weeks post-OVX has been reported at other skeletal sites such as the femur and tibia, we used 12 weeks post-OVX as a way to group studies included in this perspective (4, 11, 18).

All studies measuring BMD of the whole mandible by DXA at ≥12 weeks post-OVX reported no significant differences in BMD between OVX and SHAM rats (Supplementary Table S1). In a study of 3-month-old Sprague-Dawley rats that measured whole mandible BMD *in vivo* by DXA at several time points, BMD in OVX rats was significantly higher at week 4 than in SHAM rats, but was similar at weeks 12 and 20 (45). In a study of 4-month-old Wistar rats sacrificed at 9 weeks post-OVX, there was no significant difference in whole mandible BMD between SHAM and OVX rats (34). These results may support the observation that the hyperphagia that occurs in the first few weeks following OVX surgery may stimulate bone formation in the mandible (though food intake was not measured).

Studies of the alveolar region of the mandible demonstrate a loss of BMD and deterioration of bone structure in OVX rats regardless of whether duration of time since OVX surgery was <12 weeks or ≥12 weeks (Supplementary Table S1). For the mandibular body, most studies with a post-OVX period of ≥12 weeks found significantly lower BMD and deterioration of bone structure in OVX compared to SHAM rats, while most studies with a post-OVX period of <12 weeks did not find significant differences. These results are similar to the pattern seen in the whole mandible, likely because both regions are comprised of both trabecular and cortical bone and would respond similarly to OVX. In the mandibular condyle, most studies with a post-OVX period of ≥12 weeks found significantly lower BMD and deterioration of bone structure. Interestingly, two studies that analyzed bone outcomes in 17-week-old rats over several time points in the condyle for <12 weeks post-surgery found that some bone parameters such as BMD and BV/TV increased in SHAM rats over time, suggesting that growth was still occurring in this region (46, 47). Findings in the cortical bone of the mandible with a study duration <12 weeks post-OVX showed effects less consistent with OVX, including increased mineral-to-matrix ratio and tissue mineral density OVX compared to SHAM rats at week 8. Studies with time points of ≥12 weeks post-OVX (up to 52 weeks) mostly found a lower BMD or BMC of cortical bone as well as decreased cortical thickness in the mandible in OVX compared to SHAM rats, especially with longer study durations. These findings support that growth in young rats and age-related bone loss may interfere with expected OVX effects.

Collectively these studies suggest that the duration of the post-OVX period should be several weeks beyond the period of hyperphagia (and in turn stimulation of mandible bone formation) that occurs over the first few weeks post-OVX while avoiding a study length in which age-related bone loss occurs. A duration of 12 weeks post-surgery may be ideal (Figure 1).

### Feeding protocol

As discussed in the previous section, the feeding protocol used (i.e., *ad libitum* or pair-feeding) is important to consider since hyperphagia that occurs post-OVX can stimulate bone formation in the mandible (28, 44). *Ad libitum* feeding refers to a method of feeding in which the diet is always available to the rats, whereas pair-feeding involves feeding rats from two different experimental groups the same amount (28). With pair-feeding, food intake of OVX rats is matched to SHAM (42). However, since OVX surgery induces hyperphagia and weight gain, pair-feeding would restrict food intake and prevent typical weight gain seen in OVX rats (42). This might be useful for controlling bone formation in the mandible of OVX rats caused by increased mechanical load from increased feeding post-OVX. However, pair-fed OVX rats may experience physiological and psychological stress due to food deprivation, which may affect fluid intake and metabolic outcomes (48). After 12 h without access to food, rats efficiently reduce further fluid and energy loss through adjustments in their behaviour and physiology (48). These adjustments in food or fluid intake in OVX rats may be problematic as they could confound the results of a nutritional intervention. As stated earlier, with *ad libitum* feeding, OVX rats often have higher food intake compared to SHAM rats, resulting in an increase in mastication force and increased mechanical repetitive load on the mandible, which stimulates bone formation (34).

Most studies analyzing the mandible and maxilla in the OVX rat model use the *ad libitum* feeding protocol or do not report what feeding protocol was used (Supplementary Tables S1 and S2). Lack of reporting of this aspect of study design makes it difficult for researchers to mimic previous study designs. However, among the four studies of the whole mandible that reported feeding protocol, all studies found a non-significant difference in BMD between OVX and SHAM rats, regardless of whether rats were fed *ad libitum* or pair-fed. In the alveolar bone region of the mandible, all except two studies using an *ad libitum* feeding protocol found significantly lower BMD and bone structure parameters in OVX rats compared to SHAM rats, whereas all four studies that used pair-feeding demonstrated this same result. One study of 6-month-old rats compared bone structure (BV/TV, BS/BV, Tb.Th, Tb.N, and Tb.Sp) in the alveolar bone and condyle of the mandible in two experimental OVX groups fed either *ad libitum* or pair-fed to the SHAM rats (28). There was a significant decrease in BV/TV, BS/BV, and Tb.N and a significant increase in Tb.Sp in OVX rats at week 16 and 36 post-surgery compared to SHAM rats (28). While there was no significant difference in bone structure parameters between the two OVX groups, there was a trend towards greater deterioration of bone structure in pair-fed OVX rats, which may suggest that pair-feeding further challenges bone structure in the mandible in OVX rats (28).

In summary, researchers are encouraged to report whether *ad libitum* or pair-feeding was used to facilitate comparisons of OVX studies with similar study design and improve heterogeneity in future SRs and meta-analyses (Figure 1). Just as food consumption is measured in studies utilizing a pair-feeding protocol, studies in rats fed *ad libitum* should also report weekly food intake in OVX and SHAM rats to highlight the occurrence and timing of OVX-induced hyperphagia which may influence bone outcomes. It is recognized that measuring food intake takes considerable time and it may be more practical for investigators to report that feeding was *ad libitum* if it is not feasible to measure food intake.

#### Diet texture and nutrient composition

Studies should aim to report as many details of the diet as possible. This includes diet texture, such as whether the diet is in the form of a pellet or powder. Pelleted diets are more widely used, while powdered diets may be used if a component is added into the diet within an individual lab setting (i.e., a polyphenol extract). A pelleted diet introduces increased mechanical loading on the mandible and maxilla during chewing, which may stimulate bone formation at these skeletal sites. In contrast, powdered diets have been used to determine the effects of masticatory hypofunction on the mandible in OVX rats, which lead to a loss of BMD and deterioration of bone structure compared to rats that consumed a pelleted diet (34, 49). Studies should also report whether the diet is a standard rat chow or a semi-purified diet, such as the AIN-93M diet. A semi-purified diet is made of refined ingredients (such as isolated protein, refined sugars, and oils) and purified sources of vitamins and minerals, as opposed to ingredients entirely from natural sources (as in a standard rat chow), and leads to greater variation in diet composition (50). The nutrient composition of these diets is also important, especially the dietary levels of calcium and vitamin D in studies of bone metabolism. The current levels of calcium and vitamin D in the AIN-93M diet (5 g calcium and 1000 IU of vitamin D per kg diet) that is used widely in nutritional intervention studies were developed to support bone metabolism in rats. Despite variation in the source of ingredients in a standard rat chow, the levels of dietary calcium are quite consistent (i.e., ∼8 g of calcium per kg diet). It is known that lowering the dietary levels of calcium and vitamin D results in a loss of BMD and deterioration of bone structure in rats and exacerbates the effects of OVX (37, 51). Thus, reporting details on diet composition allows researchers to utilize the same diet and nutrient intake used in previous studies. This aids with reproducibility and facilitates comparisons of outcomes across different interventions.

Most studies reported whether the diet used was a standard rat chow or a purified diet, however, the nutrient composition was often not specified (Supplementary Tables S1 and S2). Although standard rat chows, in general, have higher levels of calcium per kg diet than the AIN-93M control diet, studies that utilized a standard rat chow did observe a loss of BMD and deterioration of bone structure in OVX rats. Thus, it appears that higher levels of dietary calcium did not prevent OVX effects. In contrast, calcium-deficient diets (0.05–1.0 g of calcium per kg diet) led to further decreases in BMD, BMC, and/or bone volume at different sites of the mandible and maxilla in OVX rats (37–39, 51, 52). Despite the large proportion of studies that left diet unreported, OVX was shown to affect bone outcomes in these studies. To summarize, rodent diets can be highly variable and contribute to heterogeneity among studies. Researchers should report as many details as possible about the diet used, including the brand name and code (where possible), diet texture, and nutrient composition, to facilitate systematic comparisons of studies (Figure 1).

### Type of imaging

The two most widely used types of imaging for measuring bone metabolism in these studies included dual-energy x-ray absorptiometry (DXA) and micro-computed tomography (micro-CT). BMD can be measured using DXA or micro-CT, such that BMD is an areal or volumetric measurement, respectively. Micro-CT can also be used to measure bone structure, including bone volume fraction (BV/TV) and trabecular thickness, separation, and number (Tb.Th, Tb.Sp, Tb.N) and thus provides a more comprehensive assessment of bone health at a given skeletal site. DXA is generally more available to researchers and provides an almost instantaneous measurement of BMD while obtaining bone structure measures involves multiple steps. However, the ability to obtain a more comprehensive assessment of bone using micro-CT is advantageous. As noted in current guidelines, it is essential to report specific details: the model and manufacturer of the micro-CT, scan settings (voxel size, voltage, electrical current, exposure time), region/volume of interest, reconstruction settings and image analysis software, and specific bone structure parameters (53). Most studies using micro-CT reported these details. In summary, using micro-CT for analyzing changes in bone structure due to intervention in OVX rats is ideal as it provides a comprehensive assessment of BMD and bone structure (Figure 1).

## Conclusion

Following the recommendations in Figure 1—and careful reporting of study design details that aligns with the ARRIVE guidelines—will help reduce heterogeneity among future studies, and, ultimately, allow SRs with meta-analyses to form reliable conclusions about the effect of OVX on outcomes measured in mandible and maxilla. A comprehensive understanding of the response to OVX is valuable for investigators planning and conducting interventions using this model.
