# Supplementary material for: Study design considerations for mandibular and maxillary research in the OVX rat model
Source: Front Dent Med. 2026 Oct 27;7:1968183. doi: 10.3389/fdmed.2026.1968183 (PMC13748403; doi:10.3389/fdmed.2026.1968183)
Supplement: Supplementary file 1 [file Table1.docx]

**Supplementary Table 1**. Changes in bone mineral density and bone structure in the mandible in rats post-ovariectomy.

| **Study,**  **Rat strain, Age at OVX,**  **Study duration (Measurement time)** | **Diet composition** | **Feeding protocol** | **Diet texture** | **Outcomes measured (Imaging used)** | **Region(s) of interest** | **Results**  OVX vs. SHAM |
| --- | --- | --- | --- | --- | --- | --- |
| **Mandible** | | | | | | |
| **Whole mandible** | | | | | | |
| Orrico et al., 2007 [^1^]  Holtzman, 12.9 weeks,  21.4 weeks  (week 7.3 and 21.4) | NR | Ad libitum | NR | BMD (DXA) | Entire mandible | 1. NS difference at both time points. 2. Age-related bone growth in SHAM at week 21.4 and OVX at both time points. |
| Patullo et al., 2009 [^2^]  Wistar, 16 weeks,  9 weeks (week 9) | Standard rat diet | Ad libitum | Hard (pellet) and soft (powder) | BMD (DXA) | Entire mandible excluding molar and incisors (crown) | 1. NS difference (trend: higher BMD in OVX rats). 2. Significantly lower in soft-diet groups compared to hard-diet groups. |
| Elovic et al., 1995 [^3^]  Sprague-Dawley, 24 weeks,  28.6 weeks  (week 16.3 and 28.6) | Standard rat diet, 10 g Ca/kg diet | Ad libitum | NR | BMD (DXA) and bone area fraction (stereology) | Entire mandible | 1. NS difference in BMD at both time points. 2. Age-related increase in BMD at both time points. 3. NS difference in bone area fraction at week 16.3. 4. Significantly lower bone area fraction in OVX at wk 28.6 compared to SHAM and baseline OVX (aging). |
| Kuroda et al., 2003 [^4^]  Sprague-Dawley, 13 weeks,  15.6 weeks (week 15.6) | NR | Pair-feeding | NR | BMD  (DXA) | Entire mandible | 1. NS difference. |
| Yu et al., 2012 [^5^]  Sprague-Dawley, 12 weeks,  20 weeks  (week 4, 12, and 20) | NR | NR | NR | BMD,  (DXA, *in vivo*) | Entire mandible | 1. Significantly higher BMD in OVX rats at week 4. 2. NS difference at weeks 12 and 20 (trend: lower BMD in OVX rats at week 12). |
| **Alveolar bone (molar region)** | | | | | | |
| Moriya et al., 1998 [^6^]  Sprague-Dawley, 4 weeks,  4 weeks (week 4) | Standard rat diet, 8.92 g Ca/kg diet; and Ca-deficient diet, 0.05 g Ca/kg diet. | Ad libitum | NR | BMD (Soft X-ray photographs) | Alveolar bone surrounding M1, M2, and M3 | 1. NS difference. 2. Combined effects of OVX and Ca-deficient diet led to significantly lower BMD. |
| Liu et al., 2015 [^7^]  Wistar, 8 weeks,  12 weeks (week 12) | Standard rat diet | Ad libitum | NR | BMD, PoV/TV, Po.Dm., and Po.Sp.  (Micro-CT) | Buccal alveolar bone proper of the mesial root of M1 | 1. Significantly lower BMD and Po.Sp and significantly higher PoV/TV (porosity) in OVX. 2. NS difference in Po.Dm. |
| Chen et al., 2017 [^8^]  Wistar, 8 weeks,  20 weeks (week 20) | Standard rat diet | Ad libitum | NR | BV/TV, Tb.Th, Tb.N, and Tb.Sp (Micro-CT) | Region below M1 | 1. Significantly lower BV/TV and Tb.Th in OVX. 2. NS difference in Tb.N and Tb.Sp. |
| Rawlinson et al., 2009 [^9^]  Sprague-Dawley, 8 weeks,  20 weeks (week 20) | NR | Ad libitum | NR | Mineraliz-ation density (Micro-CT) | Frontal cross section through middle of M2 | 1. Significantly lower in OVX. |
| Longo & Ward, 2016 [^10^]  Sprague-Dawley, 12 weeks,  12 weeks (week 12) | Modified AIN-93M diet (18% kcal from protein, 56% kcal from carbo-hydrates, 25% kcal from fat; *n*-3 poly-unsaturated fatty acids source: flaxseed oil or menhaden oil) | Ad libitum | NR | BV/TV, Tb.N, Tb.Sp, and Conn.D (Micro-CT) | M1 inter-radicular septum | 1. Significantly reduced BV/TV, Tb.N, and Conn.D. 2. Significantly increased Tb.Sp in OVX. |
| Li et al., 2014 [^11^]  Sprague-Dawley, 12 weeks,  18 weeks (week 18) | Standard rat diet | Ad libitum | NR | BMD, BV/TV, Tb.Th, Tb.N, and Tb.Sp  (Micro-CT) | M1 inter-radicular septum | 1. Significantly lower BMD, BV/TV, and Tb.Th in OVX. 2. Significantly increased Tb.Sp in OVX. |
| Lee et al., 2019 [^12^]  Sprague-Dawley, 12 weeks,  36 weeks  (week 8, 12, 26, and 36) | Standard rat diet | Ad libitum | NR | BMD, BV/TV, Tb.Th, Tb.N, and Tb.Sp (Micro-CT) | M1 inter-radicular septum | 1. Significantly lower BMD and Tb.Th at weeks 26 and 36 and lower BV/TV at week 26 in OVX. 2. NS difference in Tb.N and Tb.Sp at all time points. |
| Esteves et al., 2015 [^13^]  Wistar, 12.9 weeks,  17.1 weeks  (week 8.6, 12.9, and 17.1) | NR | Ad libitum | NR | % bone matrix area  (Histomorph-ometry) | M1 inter-radicular septum | 1. NS difference in bone matrix area at all time points. |
| Elovic et al., 1995 [^3^]  Sprague-Dawley, 24 weeks,  28.6 weeks  (week 16.3 and 28.6) | Standard rat diet, 10 g Ca/kg diet | Ad libitum | NR | BMD  (DXA) and bone area fraction (stereology) | Molar region | 1. NS difference in BMD at both time points. 2. Age-related increase in BMD at both time points. 3. Significantly greater bone area fraction in OVX compared to SHAM at baseline. |
| Coutel et al., 2019 [^14^]  Sprague-Dawley, 24 weeks,  36 weeks (week 16 and 36) | Standard rat diet | Ad libitum | NR | BV/TV, BS/BV, Tb.Th, Tb.N, and Tb.Sp (Micro-CT) | M1 inter-radicular septum | 1. Significantly lower BV/TV, BS/BV, and Tb.N in OVX at both time points. 2. NS difference in Tb.Th at both time points. 3. Significantly increased Tb.Sp in OVX at both time points. 4. No age-related changes in bone at either time point. |
| Tanaka et al., 2003 [^15^]  Fischer, 24 weeks,  52 weeks (week 52) | Standard rat diet, 11.5 g Ca, 8.8 g P, and 800 IU vit D per kg diet | Ad libitum | NR | BV/TV, Tb.Th, Tb.N, and Tb.Sp (Micro-CT) | M1 inter-radicular septum | 1. Significantly lower BV/TV, Tb.Th, and Tb.N in OVX. 2. Significantly increased Tb.Sp in OVX. 3. Significantly increased BV/TV and Tb.Th in SHAM compared to baseline (day 0). |
| Chatterjee et al., 2021 [^16^]  Wistar, 12 weeks,  8 weeks (week 8) | Standard rat diet, 10 g Ca and 7 g P per kg diet | Pair-feeding | NR | BV/TV, Tb.Th, Tb.N, and Tb.Sp (Micro-CT) | Region surrounding M1, M2, and M3 | 1. Significantly lower BV/TV and significantly increased Tb.Sp in buccal region of M1 in OVX. NS difference in all other parameters in this region. 2. NS difference in all parameters in inter-radicular septa of M2 and M3 and lingual region of M1. |
| Duarte et al., 2006 [^17^]  Wistar, 12.9 weeks,  11.4 weeks (week 11.4) | NR | Pair-feeding | NR | Bone density (histomorph-ometry) | M1 inter-radicular septum | 1. Significantly lower bone density in OVX. |
| Kuroda et al., 2003 [^4^]  Sprague-Dawley, 13 weeks,  15.6 weeks (week 15.6) | NR | Pair-feeding | NR | Trabecular BMD (pQCT) | Mesial surface of M1 to distal surface of M3 | 1. Significantly lower at some slices in OVX. |
| Coutel et al., 2019 [^14^]  Sprague-Dawley, 24 weeks,  36 weeks (week 16 and 36) | Standard rat diet | Pair-feeding | NR | BV/TV, BS/BV, Tb.Th, Tb.N, and Tb.Sp (Micro-CT) | M1 inter-radicular septum | 1. Significantly lower BV/TV, BS/BV, and Tb.N in OVX at both time points. 2. NS difference in Tb.Th at both time points. 3. Significantly increased Tb.Sp in OVX at both time points. 4. No age-related changes in bone at either time point. |
| Liu et al., 2015 [^18^]  Wistar, 12 weeks,  20 weeks (week 20) | Standard rat diet | NR | NR | BMD, BV/TV, Tb.Th, Tb.N, and Tb.Sp  (Micro-CT) | M1 inter-radicular septum | 1. Significantly lower BMD, BV/TV and Tb.Th in OVX. 2. Significantly increased Tb.Sp in OVX. 3. NS difference in Tb.N. |
| Tanaka et al., 2002 [^19^]  Fischer, 17.1 weeks,  8.6 weeks (week 8.6) | NR | NR | NR | BV/TV, Tb.Th, Tb.N, and Tb.Sp (Histomorph-ometry) | M1 inter-radicular septum | 1. Significantly lower BV/TV and Tb.N in OVX. 2. NS difference in Tb.Th. 3. Significantly increased Tb.Sp in OVX. |
| Watanabe et al., 2020 [^20^]  Sprague-Dawley, 24 weeks,  8 weeks (week 8) | NR | NR | NR | TMD, BV/TV, BS/BV, Tb.Th, Tb.N, and Tb.Sp (Micro-CT) | Region surrounding all three molars | 1. Significantly higher TMD in whole bone region (alveolar, trabecular, and cortical bone regions combined). 2. NS difference in TMD in alveolar bone and trabecular bone region. 3. NS difference in BV/TV, BS/BV, Tb.Th, Tb.N, or Tb.Sp. |
| Liu et al., 2015 [^21^]  Sprague-Dawley, 24 weeks,  36 weeks  (week 2, 4, 12, 24, and 36) | NR | NR | NR | BMD, BV/TV, Tb.Th, Tb.N, and Tb.Sp (Micro-CT) | M1 inter-radicular septum | 1. Significantly lower BMD in OVX at weeks 24 and 36. 2. Temporal fluctuation in BV/TV, Tb.Th, and Tb.N from week 0 to 4 after OVX, followed by a relatively plateau phase. 3. NS difference in BV/TV and Tb.N at all time points. 4. Significantly lower Tb.Th in OVX at week 24. 5. Significantly higher Tb.Sp in OVX at week 36. |
| **Mandibular body** | | | | | | |
| Hara et al., 2001 [^22^]  Wistar, 6 weeks,  12 weeks (week 12) | Control diet (11.5 g Ca and 3.5 g P per kg diet) and Ca-deficient diet (0.2 g Ca and 3.5 g P per kg diet) | Ad libitum | NR | Bone volume (histomorph-ometry) | Coronal cross section through M1; sampled area between molar roots and mandibular canal | 1. Significantly lower in OVX. 2. Significantly lower in rats fed Ca-deficient diet compared to control diet. |
| Hsu et al., 2016 [^23^]  Wistar, 8 weeks,  12 weeks (week 12) | Standard rat chow | Ad libitum | NR | BV/TV, Tb.Th, Tb.N, and Tb.Sp (Micro-CT) | Trabecular bone in region below M1 | 1. Significantly lower BV/TV and Tb.Th in OVX. 2. NS difference in Tb.N. 3. Significantly increased Tb.Sp in OVX. |
| Yang et al., 2003 [^24^]  Lewis-Brown-Norway, 8-12 weeks (rats age-matched),  16 weeks (week 16) | Standard rat chow | Ad libitum | NR | BV/TV, Tb.Th, and Tb.Sp (Micro-CT) | Below apex of mesial root of M1 | 1. Significantly lower BV/TV and Tb.Th in OVX. 2. Significantly increased Tb.Sp in OVX. |
| Lee et al., 2019 [^12^]  Sprague-Dawley, 12 weeks,  36 weeks  (week 8, 12, 26, and 36) | Standard rat diet | Ad libitum | NR | BMD, BV/TV, Tb.Th, Tb.N, and Tb.Sp (Micro-CT) | Region below molars from M1 to M2; excludes cortical bone | 1. Significantly lower BMD, BV/TV, and Tb.Th in OVX at week 36. 2. NS difference in Tb.N and Tb.Sp at all time points. |
| Elovic et al., 1994 [^25^]  Sprague-Dawley, 12.9 weeks,  5 weeks (week 5) | Standard rat diet, 10 g Ca/kg diet | Ad libitum | NR | BMD (DXA) and bone area fraction (stereology) | Mesial line angle of M1 to posterior of distal root of M3 | 1. NS difference in BMD or bone area fraction. |
| Irie et al., 2004 [^26^]  Wistar, 25 weeks,  9 weeks (week 9) | 11.7 g Ca, 9.1 g P, and 800 IU vit D per kg diet | Ad libitum | NR | BV/TV, Tb.Th, Tb.N, and Tb.Sp (Micro-CT) | Between roots of molar and root of incisor | 1. Significantly lower BV/TV and Tb.Th in OVX. 2. NS difference in Tb.N. 3. Significantly increased Tb.Sp in OVX. |
| Kuroda et al., 2003 [^4^]  Sprague-Dawley, 13 weeks,  15.6 weeks (week 15.6) | NR | Pair-feeding | NR | Cortical BMD (pQCT) | Mesial surface of M1 to distal surface of M3; excluding incisor and molars | 1. NS difference. |
| Mavropoulos et al., 2007 [^27^]  Sprague-Dawley, 24 weeks,  16 weeks (week 16) | Diet with 15% casein, 80% carbohydrates, 5% fat, and 11 g Ca, 8 g P, 2 g Mg per kg diet; daily dose of 100 IU vit D/kg body weight dissolved in peanut oil | Pair-feeding | NR | BMD (DXA); BV/TV, BS/BV, Tb.Th, Tb.N, and Conn.D (Micro-CT) | Alveolar process (DXA) and alveolar bone between roots of molars and root of incisor (Micro-CT) | 1. NS difference in BMD, BV/TV, BS/BV, or Tb.Th. 2. Significant reduction in Conn.D and Tb.N in OVX. |
| Mavropoulos et al., 2014 [^28^]  Sprague-Dawley, 24 weeks,  16 weeks (week 16) | NR | Pair-feeding | Hard (pellet) or soft (viscid) | BV/TV, Tb.Th, Tb.N, Tb.Sp, and Conn.D (Micro-CT) | Alveolar process | 1. NS difference in BV/TV, Tb.Th, and Conn.D. 2. Significantly lower Tb.N and significantly higher Tb.Sp in OVX. 3. Soft diet led to a further decrease of BV/TV, Tb.Th, and Tb.N, and increase in Tb.Sp. |
| Watanabe et al., 2020 [^20^]  Sprague-Dawley, 24 weeks,  8 weeks (week 8) | NR | NR | NR | BMD (Micro-CT) | Regions surrounding M1, M2, M3 | 1. NS difference. |
| Jiang et al., 2008 [^29^]  Wistar, 26 weeks,  16 weeks (week 16) | Modified AIN-93G diet (1 g Ca/kg diet) for SHAM and OVX, vs. basal controls (10 g Ca/kg diet) | NR | NR | Total BMD (trabecular and cortical) (pQCT) | One coronal slice through M1 | 1. Significantly lower in OVX. 2. Modified AIN-93G diet led to significantly lower total BMD in SHAM compared to basal control. Effect was enhanced by OVX. |
| Jiang et al., 2004 [^30^]  Wistar, 26 weeks,  16 weeks (week 16) | Ca-deficient diet (1g Ca/kg diet) for SHAM and OVX, vs. basal controls (10 g Ca/kg diet) | NR | NR | Trabecular BMD and BMC (pQCT) | 11 coronal sections from M1 (mesial margin) to M3 (distal margin) | 1. Significantly lower BMD (all sites) and BMC (at 3 out of 11 sites, from M2 mesial border to centre of M1) in OVX. 2. Ca-deficient diet led to significantly lower BMD (at 4 out of 11 sites) and BMC (at 8 out of 11 sites, mostly beneath M1). |
| Jiang et al., 2003 [^31^]  Wistar, 35 weeks,  24 weeks (week 12 and 24) | Modified AIN-93G diet (0.1 g Ca/kg diet) for SHAM and OVX, vs. basal controls (10 g Ca/kg diet) | NR | NR | Trabecular BMD and BMC (pQCT) | Mesial surface of M1 to distal surface of M2 | 1. Significantly lower trabecular and total (trabecular and cortical) BMD and BMC in OVX at both time points. 2. Modified AIN-93G diet led to a significantly lower total and trabecular BMD and BMC at both time points. Effect was enhanced by OVX. |
| Sun et al., 2014 [^32^]  Sprague-Dawley, NR,  12 weeks (week 12) | NR | NR | NR | BV/TV, Tb.Th, Tb.N, Tb.Sp, and Conn.D (Micro-CT) | Alveolar bone below roots of M1 | 1. Significantly lower BV/TV, Tb.Th, Tb.N, and Conn.D in OVX. 2. Significantly increased Tb.Sp in OVX. |
| Liu et al., 2015 [^33^]  Sprague-Dawley, NR,  12 weeks (week 12) | NR | NR | NR | BMD (Micro-CT) | M1, M2, M3 | 1. NS difference. |
| **Condyle** | | | | | | |
| Lee et al., 2019 [^12^]  Sprague-Dawley, 12 weeks,  36 weeks  (week 8, 12, 26, and 36) | Standard rat diet | Ad libitum | NR | BMD, BV/TV, Tb.Th, Tb.N, and Tb.Sp (Micro-CT) | Subchondral and central regions | 1. NS difference in all trabecular bone parameters in either the subchondral or central region. 2. Significantly lower BMD in central region of condyle in OVX at week 36 only. |
| Tanaka et al., 1998 [^34^]  Fischer, 17.1 weeks,  8.6 weeks  (week 1, 2, 4.3, and 8.6) | Standard rat diet | Ad libitum | NR | BMD (DXA) and bone marrow area (soft X-ray photography) | Mandibular condyle (above straight line between the two most depressed points) | 1. NS difference in BMD. 2. In both groups, BMD gradually increased with time. 3. Significantly higher bone marrow area in OVX from week 2 and onward. 4. Age-related changes (i.e., narrowing) in bone marrow area in SHAM at week 2 and week 4.3. |
| Tanaka et al., 1999 [^35^]  Fischer, 17.1 weeks,  8.6 weeks  (week 1, 2, 4.3, and 8.6) | Standard rat diet | Ad libitum | NR | BV/TV, Tb.Th, Tb.N, and Tb.Sp (Histomorph-ometry) | Subchondral and central regions | 1. In subchondral and central regions, BV/TV significantly increased with time in SHAM. 2. In subchondral region, BV/TV in OVX decreased significantly at week 1 then recovered to reach same as SHAM from week 2 onward. 3. In subchondral region, Tb.Th significantly increased with time in both groups. 4. In subchondral region, Tb.N and Tb.Sp significantly decreased with time in SHAM. Tb.N in subchondral region decreased gradually in OVX in similar pattern to SHAM. 5. In subchondral region, Tb.Th was significantly lower in OVX. 6. Significantly lower BV/TV in OVX in central region at week 8.6. No significant changes with time. 7. Significantly increased Tb.Sp in OVX in subchondral and central regions. 8. In central region, Tb.Th significantly increased Tb.N significantly decreased after week 2 in both groups. 9. Unchanged Tb.Sp throughout experiment in SHAM. |
| Patullo et al., 2009 [^2^]  Wistar, 16 weeks,  9 weeks (week 9) | Standard rat diet | Ad libitum | Hard (pellet) and Soft (powder) | BV/TV, Tb.Th, Tb.N, and Tb.Sp (Histomorph-ometry) | Greatest convexity of condylar process | 1. NS difference. 2. Significantly lower BV/TV and Tb.Th in soft diet group compared with hard diet. |
| Jiang et al., 2017 [^36^]  Sprague-Dawley, 24 weeks,  12 weeks (week 12) | NR | Ad libitum | NR | BV/TV, Tb.Th, and Tb.Sp (Micro-CT) | Region between maximum width of condyle | 1. Significantly lower BV/TV and Tb.Th in OVX. 2. Significantly increased Tb.Sp in OVX. |
| Coutel et al., 2019 [^14^]  Sprague-Dawley, 24 weeks,  36 weeks (week 16 and 36) | Standard rat diet | Ad libitum | NR | BV/TV, BS/BV, Tb.Th, Tb.N, and Tb.Sp (Micro-CT) | Central area | 1. NS difference in BV/TV at both time points. 2. Significantly lower BS/BV and Tb.N in OVX at both time points. 3. Significantly increased Tb.Th in OVX at both time points. 4. Significantly increased Tb.Sp in OVX at week 36, but NS difference at week 16. 5. No age-related changes in bone at either time point. |
| Sakakura et al., 2001 [^37^]  Wistar, 25 weeks,  9 weeks (week 9) | NR | Ad libitum | NR | Bone marrow area (Soft X-ray photography) | Neck of condylar process | 1. Enlargement of bone marrow cavity in OVX. |
| Chatterjee et al., 2021 [^16^]  Wistar, 12 weeks,  8 weeks (week 8) | Standard rat diet, 10 g Ca and 7 g P per kg diet | Pair-feeding | NR | BV/TV, Tb.Th, Tb.N, and Tb.Sp (Micro-CT) | Condylar head | 1. NS difference in all parameters. |
| Kuroda et al., 2003 [^4^]  Sprague-Dawley, 13 weeks,  15.6 weeks (week 15.6) | NR | Pair-feeding | NR | BMD (DXA) | Condylar region | 1. Significantly lower in OVX. |
| Coutel et al., 2019 [^14^]  Sprague-Dawley, 24 weeks,  36 weeks (week 16 and 36) | Standard rat diet | Pair-feeding | NR | BV/TV, BS/BV, Tb.Th, Tb.N, and Tb.Sp (Micro-CT) | Central area | 1. NS difference in BV/TV at both time points. 2. Significantly lower BS/BV and Tb.N in OVX at both time points. 3. Significantly increased Tb.Th in OVX at both time points. 4. Significantly increased Tb.Sp in OVX at week 36, but NS difference at week 16. 5. No age-related changes in bone at either time point. |
| **Cortical bone** | | | | | | |
| Hsu et al., 2016 [^23^]  Wistar, 8 weeks,  12 weeks (week 12) | Standard rat chow | Ad libitum | NR | Ct.Th (Micro-CT) | Masseteric ridge | 1. NS difference. |
| Fu et al., 2012 [^38^]  Sprague-Dawley, 12 weeks,  32 weeks (week 8, 16, and 32) | Standard rat chow (without phytoestrogens) | Ad libitum | NR | Mineral-to-matrix ratio (Raman spectro-scopy) | Buccal cortical bone | 1. Significantly greater in OVX at week 8 but significantly decreased at week 32. |
| Yang et al., 2005 [^39^]  Lewis-Brown-Norway, 12 weeks,  52 weeks (week 52) | Standard rat chow (without phytoestrogens) | Ad libitum | NR | Ct.Th  (Radiograph) | Inferior border (mean of three sites, from behind the incisor to the most posterior point of the mandibular angle) | 1. Significantly lower in OVX. |
| Watanabe et al., 2020 [^20^]  Sprague-Dawley, 24 weeks,  8 weeks (week 8) | NR | NR | NR | TMD (Micro-CT) | Cortical bone region surrounding the molars | 1. Significantly increased TMD in OVX. |
| Jiang et al., 2008 [^29^]  Wistar, 26 weeks,  16 weeks (week 16) | Modified AIN-93G diet (1 g Ca/kg diet) fed to SHAM and OVX rats; basal control rats fed diet with 10 g Ca/kg diet | NR | NR | Cortical BMD, bone area, and bone thickness (pQCT) | Frontal cross-section through M1 | 1. NS difference in cortical BMD, cortical bone area, or cortical bone thickness. 2. Modified AIN-93G diet led to significantly lower cortical BMD, cortical bone area, and cortical bone thickness in SHAM compared to basal control. Effect was not enhanced by OVX. |
| Jiang et al., 2003 [^31^]  Wistar, 35 weeks,  24 weeks (week 12 and 24) | Modified AIN-93G diet (0.1 g Ca/kg diet) fed to SHAM and OVX rats; basal control rats fed diet with 10 g Ca/kg diet | NR | NR | Cortical BMD and BMC (pQCT) | Mesial surface of M1 to distal surface of M2 | 1. Significantly lower cortical BMD and BMC in OVX at week 12. 2. NS difference in cortical BMD but significantly lower cortical BMC in OVX at week 24. 3. Modified AIN-93G diet led to significantly lower cortical BMD and BMC at both time points. These effects were enhanced by OVX, with the exception of cortical BMD at week 24. |
| **Incisor** | | | | | | |
| Kuroda et al., 2003 [^4^]  Sprague-Dawley, 13 weeks,  15.6 weeks (week 15.6) | NR | Pair-feeding | NR | BMD (DXA) | Incisal edge | 1. NS difference. |
| **SYSTEMATIC REVIEW** | | | | | | |
| Lee et al., 2019 [^40^] ^†^  Mostly Sprague-Dawley, 4-25 weeks,  N/A (1-51 weeks) | Standard rat diet | Ad libitum and/or pair-feeding | Mostly NR | BMD, BV/TV, Tb.Th, Tb.N, and/or Tb.Sp (DXA, micro-CT, pQCT, soft X-ray photography, and/or histomorph-ometry) | Mandibular body, M1 interradicular septum, mandibular condyle, and/or whole mandible | 1. Significantly lower BMD, BV/TV, and Tb.Th in OVX. 2. Significantly increased Tb.Sp in OVX. 3. Longer post-OVX periods led to greater changes in BMD. 4. The age of the rats at OVX was not linked to bone microarchitectural changes. 5. High heterogeneity was noted and attributed to the ROI studied in the mandible and the duration of the post-OVX period used. Rat strain did not lead to high heterogeneity. |

OVX, ovariectomized group; SHAM, sham-ovariectomized group; NR, not reported; BMD, bone mineral density; DXA, dual-energy X-ray absorptiometry; NS, non-significant; Ca, calcium; M1, first molar; M2, second molar; M3, third molar; PoV/TV, pore volume/total volume; Po.Dm., pore diameter; Po.Sp., pore spacing; Micro-CT, micro-computed tomography; BV/TV, bone volume fraction; Tb.Th, trabecular thickness; Tb.N, trabecular number; Tb.Sp, trabecular separation; Conn.D, connectivity density; BS/BV, bone surface fraction; P, phosphorus; vit D, vitamin D; Ct.Th, cortical thickness; pQCT, peripheral quantitative computed tomography; TMD, tissue mineral density; Mg, magnesium; BMC, bone mineral content.

^†^This systematic review and meta-analysis included the following primary studies summarized in Table 1: Patullo et al., 2009 [^2^], Kuroda et al., 2003 [^4^], Liu et al., 2015 [^7^], Li et al., 2014 [^11^], Tanaka et al., 2003 [^15^], Liu et al., 2015 [^18^], Moriya et al., 1998 [^6^], Hsu et al., 2016 [^23^], Yang et al., 2003 [^24^], Mavropoulos et al., 2014 [^28^], Sun et al., 2014 [^32^], Liu et al., 2015 [^33^], Tanaka et al., 1998 [^34^], Jiang et al., 2017 [^36^].

**Supplementary Table 2**. Changes in bone mineral density and bone structure in the maxilla in rats post-ovariectomy.

| **Study,**  **Rat strain, Age at OVX,**  **Study duration (Measurement time)** | **Diet composition** | **Feeding protocol** | **Diet texture** | **Outcomes measured (Imaging used)** | **Region(s) of interest** | **Results**  OVX vs. SHAM |
| --- | --- | --- | --- | --- | --- | --- |
| **Maxilla** | | | | | | |
| **Alveolar bone (molar region)** | | | | | | |
| Moriya et al., 1998 [^6^]  Sprague-Dawley, 4 weeks,  4 weeks (week 4) | Standard rat diet, 8.92 g Ca/kg diet; and Ca-deficient diet, 0.05 g Ca/kg diet | Ad libitum | NR | BMD  (Soft X-ray photographs) | Region surrounding the molars | 1. NS difference. 2. Combined effects of OVX and Ca-deficient diet led to significantly lower BMD. |
| Rawlinson et al., 2009 [^9^]  Sprague-Dawley, 8 weeks,  20 weeks (week 20) | NR | Ad libitum | NR | Mineraliz-ation density (Micro-CT) | Coronal cross section through M2 | 1. Significantly lower in OVX. |
| Romualdo et al., 2018 [^41^]  Wistar, 12 weeks,  12 weeks (week 12) | NR | Ad libitum | NR | BMD, BV/TV, Tb.Th, Tb.N, and Tb.Sp (Micro-CT) | M1 inter-radicular septum | 1. Significantly lower BMD in OVX. 2. NS difference in BV/TV, Tb.Th, Tb.N, or Tb.Sp. |
| Esteves et al., 2015 [^13^]  Wistar, 12.9 weeks,  17.1 weeks  (week 8.6, 12.9, and 17.1) | NR | Ad libitum | NR | % bone matrix area (Histomorph-ometry) | M1 inter-radicular septum | 1. NS difference in bone matrix area at all time points. |
| Chatterjee et al., 2021 [^16^]  Wistar, 12 weeks,  8 weeks (week 8) | Standard rat diet, 10 g Ca and 7 g P per kg diet | Pair-feeding | NR | BV/TV, Tb.Th, Tb.N, and Tb.Sp (Micro-CT) | M2 inter-radicular septum | 1. Significantly lower BV/TV in OVX |
| Dai et al., 2014 [^42^]  Sprague-Dawley, 6 weeks,  12 weeks (week 12) | NR | NR | NR | BMD, BV/TV, Tb.Th, Tb.N, and Tb.Sp (Micro-CT) | M1 inter-radicular septum | 1. Significantly lower BMD, BV/TV, and Tb.Th in OVX. 2. NS difference in Tb.N. 3. Significantly increased Tb.Sp in OVX. |
| Ishihara et al., 1999 [^43^]  Sprague-Dawley, 12 weeks,  20 weeks (week 20) | NR | NR | NR | Percent bone area (Quantitative backscattered electron microscopy) | Bucco-palatal sections through mesio-buccal and/or mesio-palatal roots of M3 | 1. NS difference. |
| Liu et al., 2015 [^21^]  Sprague-Dawley, 24 weeks,  36 weeks  (week 2, 4, 12, 24, and 36) | NR | NR | NR | BMD, BV/TV, Tb.Th, Tb.N, and Tb.Sp (Micro-CT) | M1 inter-radicular septum | 1. Significantly lower BMD in OVX at week 24. 2. Temporal fluctuation in BV/TV, Tb.Th, and Tb.N from week 0 to 4 after OVX, followed by a relatively plateau phase. 3. Significantly lower BV/TV in OVX at week 24. Slightly lower at week 36, but NS difference. 4. Significantly lower Tb.Th in OVX at weeks 2, 12, and 24. 5. Significantly increased Tb.N in OVX at week 2, but NS difference at all other time points. 6. Significantly increased Tb.Sp in OVX at week 24. |
| **Hard Palate** | | | | | | |
| Hara et al., 2001 [^22^]  Wistar, 6 weeks,  12 weeks (week 12) | Control diet (11.5 g Ca and 3.5 g P per kg diet) and Ca-deficient diet (0.2 g Ca and 3.5 g P per kg diet) | Ad libitum | NR | Bone volume (histomorph-ometry) | Coronal section through M1; sampled area between medial palatal suture and palatal groove | 1. NS difference. 2. Significantly lower in rats fed Ca-deficient diet compared to control diet. |
| **Tuberosity** | | | | | | |
| Chatterjee et al., 2021 [^16^]  Wistar, 12 weeks,  8 weeks (week 8) | Standard rat diet, 10 g Ca and 7 g P per kg diet | Pair-feeding | NR | BV/TV, Tb.Th, Tb.N, and Tb.Sp (Micro-CT) | Located on hard palate posterior to upper molars | 1. NS difference in all parameters. |

OVX, ovariectomized group; SHAM, sham-ovariectomized group; Ca, calcium; NR, not reported; BMD, bone mineral density; NS, non-significant; Micro-CT, micro-computed tomography; M2, second molar; BV/TV, bone volume fraction; Tb.Th, trabecular thickness; Tb.N, trabecular number; Tb.Sp, trabecular separation; M1, first molar; P, phosphorus; M3, third molar.

**References**

1.	Orrico SRP, Giro G, Gonçalves D, Takayama L, Pereira RMR. Influence of the Period After Ovariectomy on Femoral and Mandibular Bone Density and on Induced Periodontal Disease. *Journal of Periodontology*. 2007;78(1):164-169. doi:10.1902/jop.2007.060136

2.	Patullo I, Takayama L, Patullo R, Jorgetti V, Pereira R. Influence of ovariectomy and masticatory hypofunction on mandibular bone remodeling. *Oral Diseases*. 2009;15(8):580-586. doi:10.1111/j.1601-0825.2009.01599.x

3.	Elovic RP, Hipp JA, Hayes WC. Ovariectomy decreases the bone area fraction of the rat mandible. *Calcif Tissue Int*. 1995;56(4):305-310. doi:10.1007/BF00318051

4.	Kuroda S, Mukohyama H, Kondo H, et al. Bone mineral density of the mandible in ovariectomized rats: analyses using dual energy X-ray absorptiometry and peripheral quantitative computed tomography. *Oral Diseases*. 2003;9(1):24-28. doi:10.1034/j.1601-0825.2003.01810.x

5.	Yu SJ, Liu HC, E LL, Wang DS, Zhu GX. Proliferation and differentiation of osteoblasts from the mandible of osteoporotic rats. *Exp Biol Med (Maywood)*. 2012;237(4):395-406. doi:10.1258/ebm.2011.011217

6.	Moriya Y, Ito K, Murai S. Effects of experimental osteoporosis on alveolar bone loss in rats. *Journal of Oral Science*. 1998;40(4):171-175. doi:10.2334/josnusd.40.171

7.	Liu Z, Liu L, Kang C, Xie Q, Zhang B, Li Y. Effects of estrogen deficiency on microstructural changes in rat alveolar bone proper and periodontal ligament. *Molecular Medicine Reports*. 2015;12(3):3508-3515. doi:10.3892/mmr.2015.3891

8.	Chen YJ, Wang SP, Cheng FC, et al. Intermittent parathyroid hormone improve bone microarchitecture of the mandible and femoral head in ovariectomized rats. *BMC Musculoskelet Disord*. 2017;18:171. doi:10.1186/s12891-017-1530-4

9.	Rawlinson SCF, Boyde A, Davis GR, Howell PGT, Hughes FJ, Kingsmill VJ. Ovariectomy vs. Hypofunction: Their Effects on Rat Mandibular Bone. *J Dent Res*. 2009;88(7):615-620. doi:10.1177/0022034509340132

10.	Longo AB, Ward WE. Providing Flaxseed Oil but Not Menhaden Oil Protects against OVX Induced Bone Loss in the Mandible of Sprague-Dawley Rats. *Nutrients*. 2016;8(10):597. doi:10.3390/nu8100597

11.	Li CL, Liu XL, Cai WX, Lu WW, Zwahlen RA, Zheng LW. Effect of Ovariectomy on Stimulating Intracortical Remodeling in Rats. *Biomed Res Int*. 2014;2014:421431. doi:10.1155/2014/421431

12.	Lee C, Lee JH, Han SS, et al. Site-specific and time-course changes of postmenopausal osteoporosis in rat mandible: comparative study with femur. *Sci Rep*. 2019;9:14155. doi:10.1038/s41598-019-50554-w

13.	Esteves CM, Moraes RM, Gomes FC, Marcondes MS, Lima GMG, Anbinder AL. Ovariectomy-associated changes in interradicular septum and in tibia metaphysis in different observation periods in rats. *Pathology - Research and Practice*. 2015;211(2):125-129. doi:10.1016/j.prp.2014.11.015

14.	Coutel X, Delattre J, Marchandise P, et al. Mandibular bone is protected against microarchitectural alterations and bone marrow adipose conversion in ovariectomized rats. *Bone*. 2019;127(Complete):343-352. doi:10.1016/j.bone.2019.06.031

15.	Tanaka M, Toyooka E, Kohno S, Ozawa H, Ejiri S. Long-term changes in trabecular structure of aged rat alveolar bone after ovariectomy. *Oral Surgery, Oral Medicine, Oral Pathology, Oral Radiology & Endodontics*. 2003;95(4):495-502. doi:10.1067/moe.2003.135

16.	Chatterjee M, Faot F, Correa C, Kerckhofs J, Vandamme K. Is the Jaw Bone Micro-Structure Altered in Response to Osteoporosis and Bisphosphonate Treatment? A Micro-CT Analysis. *Int J Mol Sci*. 2021;22(12):6559. doi:10.3390/ijms22126559

17.	Duarte PM, Gonçalves P, Casati MZ, De Toledo S, Sallum EA, Nociti Jr FH. Estrogen and alendronate therapies may prevent the influence of estrogen deficiency on the tooth-supporting alveolar bone: a histometric study in rats. *Journal of Periodontal Research*. 2006;41(6):541-546. doi:10.1111/j.1600-0765.2006.00903.x

18.	Liu Z, Yan C, kang C, Zhang B, Li Y. Distributional Variations in Trabecular Architecture of the Mandibular Bone: An In Vivo Micro-CT Analysis in Rats. *PLoS One*. 2015;10(1):e0116194. doi:10.1371/journal.pone.0116194

19.	Tanaka M, Ejiri S, Toyooka E, Kohno S, Ozawa H. Effects of ovariectomy on trabecular structures of rat alveolar bone. *Journal of Periodontal Research*. 2002;37(2):161-165. doi:10.1034/j.1600-0765.2002.01601.x

20.	Watanabe K, Lewis S, Guo X, et al. Regional variations of jaw bone characteristics in an ovariectomized rat model. *Journal of the Mechanical Behavior of Biomedical Materials*. 2020;110:103952. doi:10.1016/j.jmbbm.2020.103952

21.	Liu XL, Li CL, Lu WW, Cai WX, Zheng LW. Skeletal site-specific response to ovariectomy in a rat model: change in bone density and microarchitecture. *Clinical Oral Implants Research*. 2015;26(4):392-398. doi:10.1111/clr.12360

22.	Hara T, Sato T, Oka M, Mori S, Shirai H. Effects of ovariectomy and/or dietary calcium deficiency on bone dynamics in the rat hard palate, mandible and proximal tibia. *Archives of Oral Biology*. 2001;46(5):443-451. doi:10.1016/S0003-9969(00)00135-7

23.	Hsu PY, Tsai MT, Wang SP, Chen YJ, Wu J, Hsu JT. Cortical Bone Morphological and Trabecular Bone Microarchitectural Changes in the Mandible and Femoral Neck of Ovariectomized Rats. *PLoS One*. 2016;11(4):e0154367. doi:10.1371/journal.pone.0154367

24.	Yang J, Pham S, Crabbe D. Effects of oestrogen deficiency on rat mandibular and tibial microarchitecture. *Dentomaxillofacial Radiology*. 2003;32(4):247-251. doi:10.1259/dmfr/12560890

25.	Elovic RP, Hipp JA, Hayes WC. Maxillary molar extraction causes increased bone loss in the mandible of ovariectomized rats. *Journal of Bone and Mineral Research*. 1995;10(7):1087-1093. doi:10.1002/jbmr.5650100713

26.	Irie K, Sakakura Y, Tsuruga E, Hosokawa Y, Yajima T. Three-dimensional Changes of the Mandible and Alveolar Bone in the Ovariectomized Rat Examined by Micro-focus Computed Tomography. *Nihon Shishubyo Gakkai Kaishi*. 2004;46(4):288-293. doi:10.2329/perio.46.288

27.	Mavropoulos A, Rizzoli R, Ammann P. Different Responsiveness of Alveolar and Tibial Bone to Bone Loss Stimuli. *Journal of Bone and Mineral Research*. 2007;22(3):403-410. doi:10.1359/jbmr.061208

28.	Mavropoulos A, Kiliaridis S, Rizzoli R, Ammann P. Normal masticatory function partially protects the rat mandibular bone from estrogen-deficiency induced osteoporosis. *Journal of Biomechanics*. 2014;47(11):2666-2671. doi:10.1016/j.jbiomech.2014.05.012

29.	Jiang GZ, Matsumoto H, Hori M, et al. Correlation among geometric, densitometric, and mechanical properties in mandible and femur of osteoporotic rats. *J Bone Miner Metab*. 2008;26(2):130-137. doi:10.1007/s00774-007-0811-7

30.	Jiang G, Matsumoto H, Yamane J, Kuboyama N, Akimoto Y, Fujii A. Prevention of trabecular bone loss in the mandible of ovariectomized rats. *J Oral Sci*. 2004;46(2):75-85. doi:10.2334/josnusd.46.75

31.	Jiang G, Matsumoto H, Fujii A. Mandible bone loss in osteoporosis rats. *J Bone Miner Metab*. 2003;21(6):388-395. doi:10.1007/s00774-003-0433-7

32.	Sun W, Wang Y qin, Yan Q, Lu R, Shi B. Effects of Er-Zhi-Wan on microarchitecture and regulation of Wnt/β-catenin signaling pathway in alveolar bone of ovariectomized rats. *Journal of Huazhong University of Science and Technology [Medical Sciences]*. 2014;34(1):114-119. doi:10.1007/s11596-014-1241-0

33.	Liu H, Gao K, Lin H, Zhang Y, Li B. Relative Skeletal Effects in Different Sites of the Mandible With the Proximal Tibia During Ovariectomy and the Subsequent Estrogen Treatment. *Journal of Oral Implantology*. 2015;41(S1):386-390. doi:10.1563/AAID-JOI-D-13-00255

34.	Tanaka M, Ejiri S, Kohno S, Ozawa H. The effect of aging and ovariectomy on mandibular condyle in rats. *The Journal of Prosthetic Dentistry*. 1998;79(6):685-690. doi:10.1016/S0022-3913(98)70076-9

35.	Tanaka M, Ejiri S, Nakajima M, Kohno S, Ozawa H. Changes of cancellous bone mass in rat mandibular condyle following ovariectomy. *Bone*. 1999;25(3):339-347. doi:10.1016/S8756-3282(99)00179-9

36.	Jiang L, Shen X, Wei L, Zhou Q, Gao Y. Effects of bisphosphonates on mandibular condyle of ovariectomized osteoporotic rats using micro‐ct and histomorphometric analysis. *J Oral Pathology Medicine*. 2017;46(5):398-404. doi:10.1111/jop.12499

37.	Sakakura Y, Shide N, Tsuruga E, Irie K, Yajima T. Effects of running exercise on the mandible and tibia of ovariectomized rats. *J Bone Miner Metab*. 2001;19(3):159-167. doi:10.1007/s007740170036

38.	Fu X, Chen J, Wu D, et al. Effects of Ovariectomy on Rat Mandibular Cortical Bone: A Study Using Raman Spectroscopy and Multivariate Analysis. *Anal Chem*. 2012;84(7):3318-3323. doi:10.1021/ac300046x

39.	Yang J, Farnell D, Devlin H, Horner K, Graham J. The effect of ovariectomy on mandibular cortical thickness in the rat. *Journal of Dentistry*. 2005;33(2):123-129. doi:10.1016/j.jdent.2004.09.001

40.	Lee JH, Han SS, Lee C, Kim YH, Battulga B. Microarchitectural changes in the mandibles of ovariectomized rats: a systematic review and meta-analysis. *BMC Oral Health*. 2019;19:128. doi:10.1186/s12903-019-0799-0

41.	Romualdo PC, Cunha NBFF, Leoni GB, et al. The effect of ovariectomy and 2 antiresorptive therapeutic agents on bone response in rats: A 3-dimensional imaging analysis. *Oral Surgery, Oral Medicine, Oral Pathology and Oral Radiology*. 2018;126(3):218-225. doi:10.1016/j.oooo.2018.04.002

42.	Dai QG, Zhang P, Wu YQ, et al. Ovariectomy induces osteoporosis in the maxillary alveolar bone: an in vivo micro-CT and histomorphometric analysis in rats. *Oral Diseases*. 2014;20(5):514-520. doi:10.1111/odi.12166

43.	Ishihara A, Sasaki T, Debari K, et al. Effects of ovariectomy on bone morphology in maxillae of mature rats. *Journal of Electron Microscopy*. 1999;48(4):465-469. doi:10.1093/oxfordjournals.jmicro.a023703
